# Effect of physiological pH on the molecular characteristics, rheological behavior, and molecular dynamics of κ-carrageenan/casein

**DOI:** 10.3389/fnut.2023.1174888

**Published:** 2023-04-14

**Authors:** Juanjuan Guo, Siliang Zhu, Peilin Chen, Zhiyu Liu, Luan Lin, Jie Zhang

**Affiliations:** ^1^College of Oceanology and Food Sciences, Quanzhou Normal University, Quanzhou, Fujian, China; ^2^Fujian Province Key Laboratory for the Development of Bioactive Material from Marine Algae, Quanzhou Normal University, Quanzhou, Fujian, China; ^3^Key Laboratory of Cultivation and High-Value Utilization of Marine Organisms in Fujian Province, Xiamen, Fujian, China; ^4^State Key Laboratory of Molecular Vaccinology and Molecular Diagnostics, School of Public Health, Xiamen University, Xiamen, China

**Keywords:** κ-carrageenan, casein, gastrointestinal pH, molecular dynamics simulation, conformational transition

## Abstract

**Introduction:**

During gastrointestinal digestion, κ-carrageenan (κ-CGN) undergoes physicochemical changes, which associated with the risk of colitis.

**Methods:**

To understand the effect of physiological pH on the conformational transition and binding stability of κ-CGN and κ-carrageenan/casein (κ-CC), we conducted experiments at pH 3.0 (gastric environment) and pH 7.0 (intestinal environment). We evaluated zeta potential, free sulfate group content, Fourier transform infrared spectroscopy, thermodynamic properties, microstructure, and molecular mechanism.

**Results and Discussion:**

Our results revealed that the helical conformation of κ-CGN and κ-CC were more ordered and stable, and sulfate group exposure both lower in the intestinal environment (pH 7.0). However, in gastric environment (pH 3.0), the charge density of κ-CGN decreased, accompanied by random curling conformation and free sulfate group content increased. In contrast, the intermolecular interactions between κ-CGN and casein increased in gastric acid environments due to casein flocculation and secondary structure folding, and significantly reduced the exposure of free sulfate groups of κ-CGN. Our research results provide an important theoretical basis for elucidating the molecular mechanism and structure-activity relationship of κ-CGN under casein matrix to protect the mucosal barrier and inhibit colitis, and are of great significance for guiding and expanding the safe application of κ-CGN, thus assisting food nutrition to be absorbed.

## 1. Introduction

Carrageenan (CGN) is a hydrophilic linear polysaccharide extracted from red algae that consists of repeating α-(1,3)-D-galactose and β-(1–4)-3,6-lactone-D-galactose units (1–3). The proportion and positions of sulfate groups and the proportion of 3,6-anhydrogalactose units differ among CGN types. These structural differences determine the different gel properties of different CGN types (4, 5). Due to its thickening and gelling properties, CGN is used as a food additive in milk, yogurt, beverages, jelly, canned foods, and sausages (6). The edible safety of κ-CGN has been controversial for a long time. A large number of animal experiments pointed out that exposure to 0.1–5% CGN solution may lead to different degrees of intestinal ulcers or intestinal inflammation, including guinea pigs, mice, rabbits, ferrets, and monkeys (7–10). However, Weiner studied the intestinal metabolism of supplemented κ-CGN (addition amount ≤ 0.1%) in infant formula, which has no significant effect on the body weight, intestinal tissue, inflammatory factors (IL-8, IL-6, and TNF-α) of piglets before weaning, and there was no intestinal inflammatory effect (11). These results drive the interest in unraveling the structure–activity relationship of κ-CGN in different vehicles and the physicochemical changes during digestion in the gastrointestinal tract.

The structure of polysaccharides affects their biological activity and solution behavior. The solution behavior of polysaccharides determines the stability of polysaccharide solutions, including aggregation, viscoelastic, and conformation transition behaviors. The coacervation in a protein-polysaccharide system is mainly affected by electrostatic interactions, pH, ionic strength, and macromolecule concentrations. Gastrointestinal digestion involves the release and absorption of nutrients from the food matrix (12). However, pH, electrolytes, and digestive enzymes may alter the molecular weight, chemical composition, structure, and conformation of polysaccharides, which are related to their biological activities (13–15). Our previous research found that κ-CGN and casein form a complex (κ-CC) *via* electrostatic interactions and undergo physical and chemical changes during simulated gastrointestinal digestion *in vitro*, which closely associated with colitis (16, 17). The gastrointestinal environment have a significant effect on the conformation and interaction between κ-CGN and casein (17).

It has been reported that κ-CGN in random coils may have more free sulfate groups and are more likely to contact the intestinal barrier mucosa, which may increase the risk of developing colonic inflammation (18, 19). We speculate that the disordered conformation and free sulfate groups of carrageenan may be the key factors leading to intestinal ulcers or intestinal inflammation. Therefore, the study of the gastrointestinal environment is crucial to assess the conformational transitions and interactions between κ-CGN and casein during digestion. In this study, we explored the effect of physiological pH on the conformation of κ-CGN and the stability of κ-CC, to provide a theoretical basis for the structure–activity relationship of carrageenan colon inflammation, which might be of great significance to guide the safe application of carrageenan, and aiding food nutrition absorption.

## 2. Materials and methods

### 2.1. Materials

We obtained κ-CGN (98% purity, 22.15% w/w sulfate content) from Solarbio Technology Co., Ltd. (Beijing, China) and bovine casein (casein sodium salt) from Sigma-Aldrich (St. Louis, MO, United States). All chemicals were of analytical grade.

### 2.2. Sample preparation

We prepared a 3% casein stock solution (w/v) by dissolving casein sodium salt in deionized water. We stirred the solution at 1,000 rpm for 2 h at room temperature. To prevent microbial contamination, we added sodium azide (50 mg·L^−1^).

We prepared a 0.5% κ-CGN solution by dissolving κ-CGN in deionized water. The solution was stirred at 800 rpm for 30 min at 60°C. A 0.5% κ-CC complex was prepared by dissolving κ-CGN in casein stock solution and stirred (800 rpm) for 30 min at 60°C. We adjusted the pH of κ-CGN and κ-CC to 2.0–8.0 using 0.01 M HCl and 0.01 M NaOH. The solutions were stored at 37°C for 2 h.

### 2.3. Zeta-potential

We determined zeta potential using a laser nanoparticle size analyzer (Zeta-sizer Nano-ZSE, Malvern Instruments Ltd., Malvern, United Kingdom) at 37°C. Each sample was measured in triplicate.

### 2.4. Sulfate group content

We measured the sulfate groups content of κ-CGN and κ-CC in solution by the turbidimetric method (20, 21). Briefly, 0.4 mL sample with 7.6 ml trichloroacetic acid was mixed and centrifuged at 8,000 rpm for 10 min. An aliquot of the supernatant (4 mL) was mixed with barium chloride-gelatin (1 mL) at room temperature for 15–20 min. We measured absorbance at 360 nm. The free sulfate group content (C_S_) of κ-CGN and κ-CC were calculated by the following equations,


$$Cs=\frac{m_{2}\times V}{m_{1}}\times 100\%$$

where $m_{1}$ is the total weight of κ-CGN powder in digestive juice (mg), $m_{2}$ is the weight of free sulfate groups exposed to the digestive juice (mg), which can be determined by the standard curve of sulfate group content (*y* = 2.0372x – 0.0016, R2 = 0.9947), and *V* is the total volume of digestive juice (mL).

### 2.5. Fourier transform infrared spectroscopy (FTIR)

We used an infrared spectrometer (Thermo Fisher Scientific, Waltham, MA, United States) to assess the chemical structure of κ-CGN and κ-CC. The freeze-dried samples (2 mg) were thoroughly mixed, ground with potassium bromide (200 mg) using a mortar pestle, and pressed into thin discs. FTIR analysis was conducted at room temperature with a spectral resolution of 4 cm^−1^, and the spectra were recorded at 4,000–400 cm^−1^.

### 2.6. Scanning electron microscopy (SEM)

The morphology of κ-CGN and κ-CC was examined by SEM (NovaNanoSEM230, FEI Instruments Ltd., United States) with an acceleration voltage of 5 kV in normal mode. We coated the freeze-dried samples with gold.

### 2.7. Rheological measurements

The dynamic rheological measurements were performed using a HER-2 Discovery rheometer (TA Instruments Ltd., United States). A parallel system with a diameter of 40 mm and a gap of 1,000 μm was used. The measurements were performed at 4°C to investigate the frequency dependence of storage and loss moduli (G′ and G″) of the κ-CGN and κ-CC gels. To minimize measurement errors, we placed the samples on the center of the plate and covered the surface with silicone oil.

#### 2.7.1. Strain sweep

The strain sweep was used to evaluate the linear viscoelastic region by determining storage modulus (G′) and loss modulus (G″), which was analyzed within the given strain value from 0.01 to 1,000% and with an angular frequency of 6.28 rad/s. A frequency sweep test was conducted at angular frequency scan range of 0.01 to 100 s^−1^ to analyze the association between angular frequency and dynamic moduli.

#### 2.7.2. Length of elastic active strands

The length of elastically active strands (L) reflects the tightness of a polymer network. Based on the Doi-Qnuki model (22), the length of elastically active strands was calculated as follows,


$$L^{3}=\frac{k_{B}T}{G_{0}}$$

where G_0_ is the low-frequency shear modulus at 0.1 Hz, k_B_ is the Boltzmann constant (1.38 × 10^−23^ J/K), and T is the absolute temperature.

### 2.8. Differential scanning calorimetry (DSC)

We used a DSC instrument (TA Instruments Ltd., United States) to record the thermal changes of κ-CGN and κ-CC in the gels. Approximately 30–50 mg of sample was transferred into an aluminum pan. The measurements were conducted in a nitrogen atmosphere. An empty aluminum pan was used as the control. The sample pan was kept at 60°C for 10 min and subsequently cooled from 60°C to 0°C at 5°C/min.

### 2.9. Molecular dynamic simulation

#### 2.9.1. Model preparation

The 2D structure of κ-CGN was obtained from PubChem databases^1^, and the energy-minimized 3D structure of κ-CGN was obtained from Maestro 11.5 with OPLS3 force field. The crystal structure of κ-casein is not included in the protein database; therefore, we used the AlphaFold2 software for homology modeling to construct κ-casein. We selected the amino acid sequence (fragment 22–190) of κ-casein in UniProt database. The constructed κ-casein model was evaluated for structural rationality by the Protein Reliability Report Module. We optimized the selected model using the OPLS3 force field of the Schrodinger Macro Model module.

The docking of κ-CC was completed with the Glide module of Schrodinger (Schrodinger, LLC: New York, NY, 2015). We calculated two docking steps including SP (standard precision) and EP (extra precision). The first step is to use the standard precision method, and the docking score scoring function is used for ranking. The output poses are set to 100. In the second step, the Rigid Receptor method is used, and the docking score scoring function is selected for scoring through high-precision EP docking. We sorted by score from high to low and output the top 20 conformations. The highest scoring binding conformation was selected; the binding mode and the interaction of κ-CGN and κ-casein were analyzed.

#### 2.9.2. Molecular dynamic simulation

We used the GPU-accelerated PMEMD program of AMBER20 software package to simulate molecular dynamics. First, we obtained the stable binding conformation by molecular docking at pH 3.0 for the residue protonation state of Protonate3D. Second, the molecular dynamics simulation was performed by Amber20 at pH 3.0, until the protein-ligand complex system equilibrated. We performed track sampling every 50 ps, and the step size was set to 0.002 ps.

### 2.10. Statistical analysis

The samples were prepared at least twice, and all experiments were performed in triplicate. We analyzed the data using SPSS software (SPSS 22.0; IBM SPSS Statistics, Chicago, IL, United States) and generated all figures with Origin Pro (V8.5, Origin Lab, Wellesley Hills, WA, United States). To determine significant differences (*p* < 0.05), we performed one-way analysis of variance.

## 3. Results and discussion

### 3.1. Zeta-potential, free sulfate group content, and macroscopic observations

**Figure 1:**
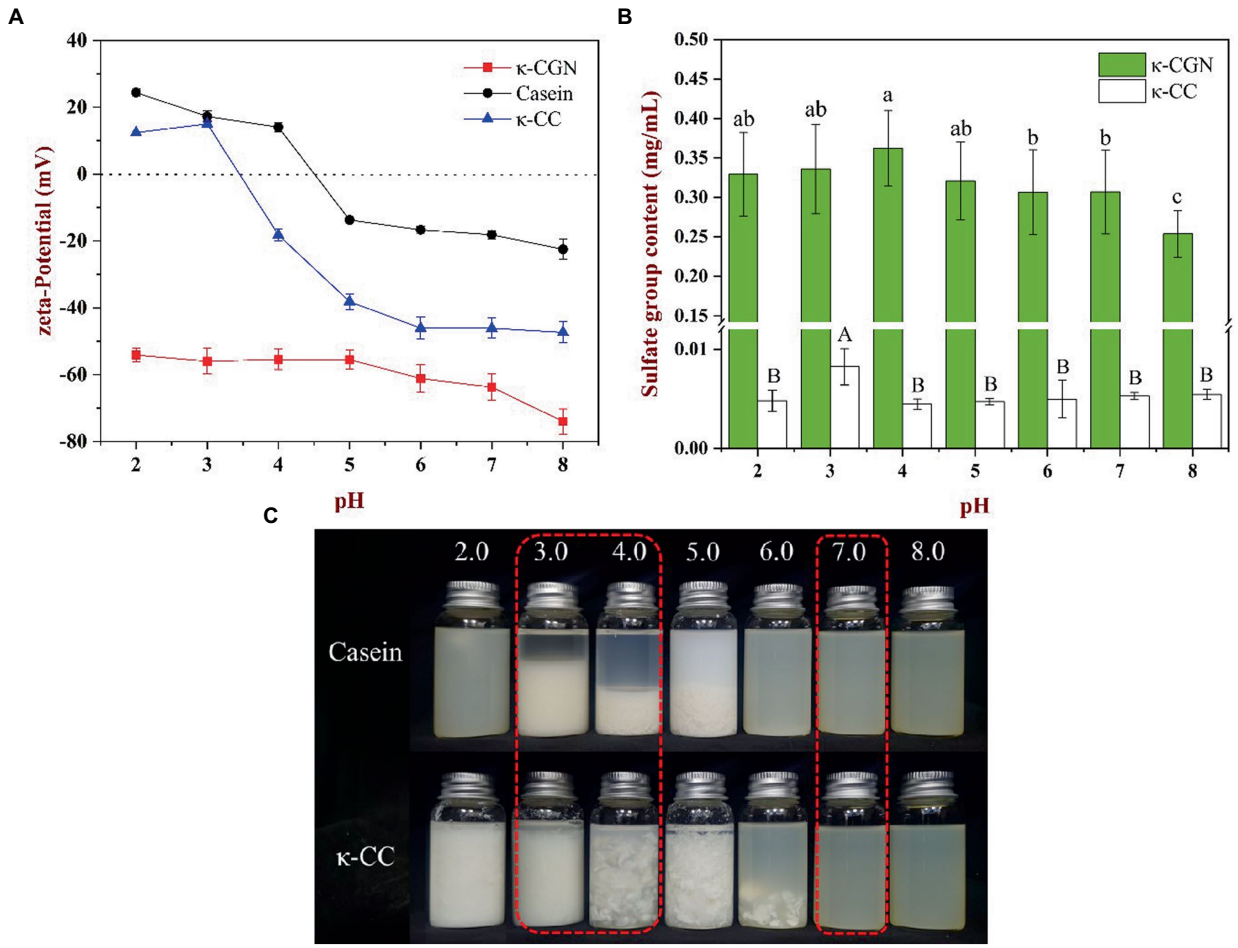
**(A)** The effect of pH on the zeta-potential of κ-CGN, casein and κ-CC complexes. **(B)** Free sulfate group content of the κ-CGN and κ-CC complexes at physiological pH in the simulated gastrointestinal phase. **(C)** Physical diagram of the κ-CGN and κ-CC complexes at physiological pH in the simulated gastrointestinal phase.

The main factor that affects the interaction between charged particles is the charge density on the surface of particles (23). Figure 1A shows the zeta potential of κ-CGN, casein, and κ-CC at different pH values. As an amphiphilic molecule, the surface charge density of protein is affected by pH. The isoelectric point (pI) of casein is 4.6. Therefore, casein is positively charged at pH < 4.6 and negatively charged at pH > 4.6. As an anionic polysaccharide, κ-CGN is negatively charged at pH 2.0 ~ 8.0. The zeta potential of κ-CGN is ~ −60 mV and increases with increasing pH. Therefore, the surface charge of polysaccharides and proteins is affected by pH. κ-CGN and casein form a complex *via* electrostatic interactions. Changes in the surface charge density of casein and κ-CGN directly affect the interaction between them (17). Furthermore, changes in the surface charge density of κ-CGN may affect the electrostatic repulsion between molecular chains, leading to changes in the stability of κ-CGN (24). Figure 1A shows that the absolute zeta potential of κ-CC decreased at pH 3.0, indicating that the charge density on the surface of κ-CC decreased, the electrostatic repulsion between molecules weakened, and the stability of the system decreased. The zeta potential of κ-CC reached an equilibrium at pH 7.0, indicating that the binding of κ-CGN to casein was more stable.

The exposure of sulfate groups in κ-CGN and κ-CC at different pH values is presented in Figure 1B. The exposure of sulfate groups in κ-CGN was the highest at pH 4.0, with a small decrease in the exposure of sulfate groups as pH decreased and increased. κ-CC consistently had a lower sulfate group exposure at pH 2.0–8.0 compared to κ-CGN. Figure 1C shows casein and κ-CC at different pH values. Casein and κ-CC precipitated at pH 3.0–5.0 and pH 2.0–6.0, respectively. The presence of κ-CGN in κ-CC inhibited the aggregation of casein particles, thereby widening the range of casein precipitation (25).

### 3.2. Fourier-transform infrared spectroscopy spectrum analysis

**Figure 2:**
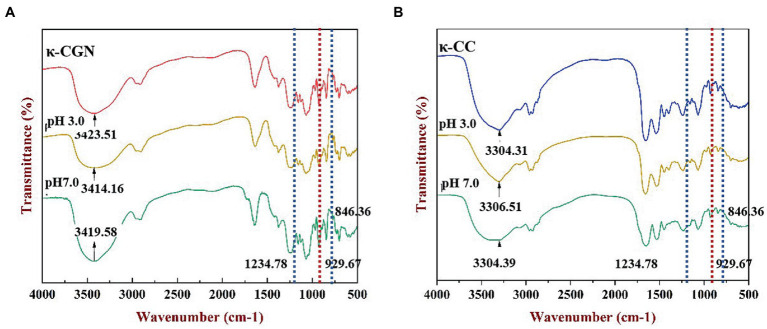
FT-IR spectra of the κ-CGN **(A)** and κ-CC **(B)** at physiological pH in the simulated gastrointestinal phase.

We used FTIR to characterize the chemical structure of κ-CGN and κ-CC (Figure 2). The broad absorption peak located near 3,423.51 cm^−1^ was attributed to the stretching vibration of O-H (Figure 2A), and the absorption peaks near 1,234.78, 929.67, and 846.36 cm^−1^ corresponded to the sulfate group (O=S=O), 3,6-anhydride-galactose, and D-galactose-4-sulfate stretching vibrations of κ-CGN (26). The absorption peak at 1,069.29 cm^−1^ represented the stretching vibration of alcoholic groups (C-OH), where the spectrum was sensitive to conformational changes in the polysaccharide backbone (27). In κ-CGN, the stretching vibrations of O-H and C-OH were significantly weaker at pH 3.0 and significantly stronger and sharper at pH 7.0, which indicated that pH has a significant effect on hydrogen bonds and molecular conformation. Casein and κ-CGN formed a stable helical complex *via* electrostatic interactions and hydrogen bonds (17, 28, 29). There were no indications of new functional groups in the spectra of κ-CC (Figure 2B), indicating that the chemical structure of κ-CC had not changed. The vibration peaks of sulfate groups and C-O single bonds weakened when pH changed, implying that changes in pH induced changes in the interactions between proteins and polysaccharides and had significant effects on the molecular conformation stability.

### 3.3. SEM analysis

**Figure 3:**
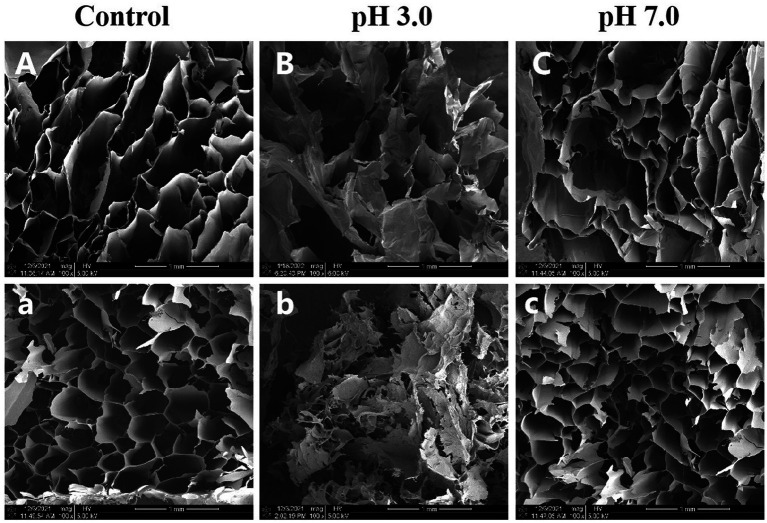
Effects of physiological pH in the simulated gastrointestinal phase on the microstructure of κ-CGN **(A–C)** and κ-CC **(a–c)** complexes.

We used SEM to investigate the effects of pH on the microstructure of κ-CGN and κ-CC during simulated gastrointestinal digestion. Figure 3A shows that κ-CGN presented a lamellar structure after gel formation, while κ-CC exhibited a relatively regular honeycomb-like network structure. This phenomenon may be due to the formation of a network structure facilitated by intermolecular entanglement caused by electrostatic interactions and hydrogen bonds between κ-CGN and casein (30). Changes in pH caused changes in the microstructure of CGN. At pH 3.0, the network of κ-CGN and κ-CC was largely disrupted (Figure 3B). In contrast, the microstructures of κ-CGN and κ-CC hardly changed at pH 7.0 (Figure 3C). This was consistent with the results obtained from FTIR, where the conformation of κ-CGN changed under the stimulus of the external environment. In acidic environments, κ-CGN became more disordered and casein lost stability, leading to a weakening in the interaction between the two molecules.

### 3.4. Rheological characterization

#### 3.4.1. Stain sweep

**Figure 4:**
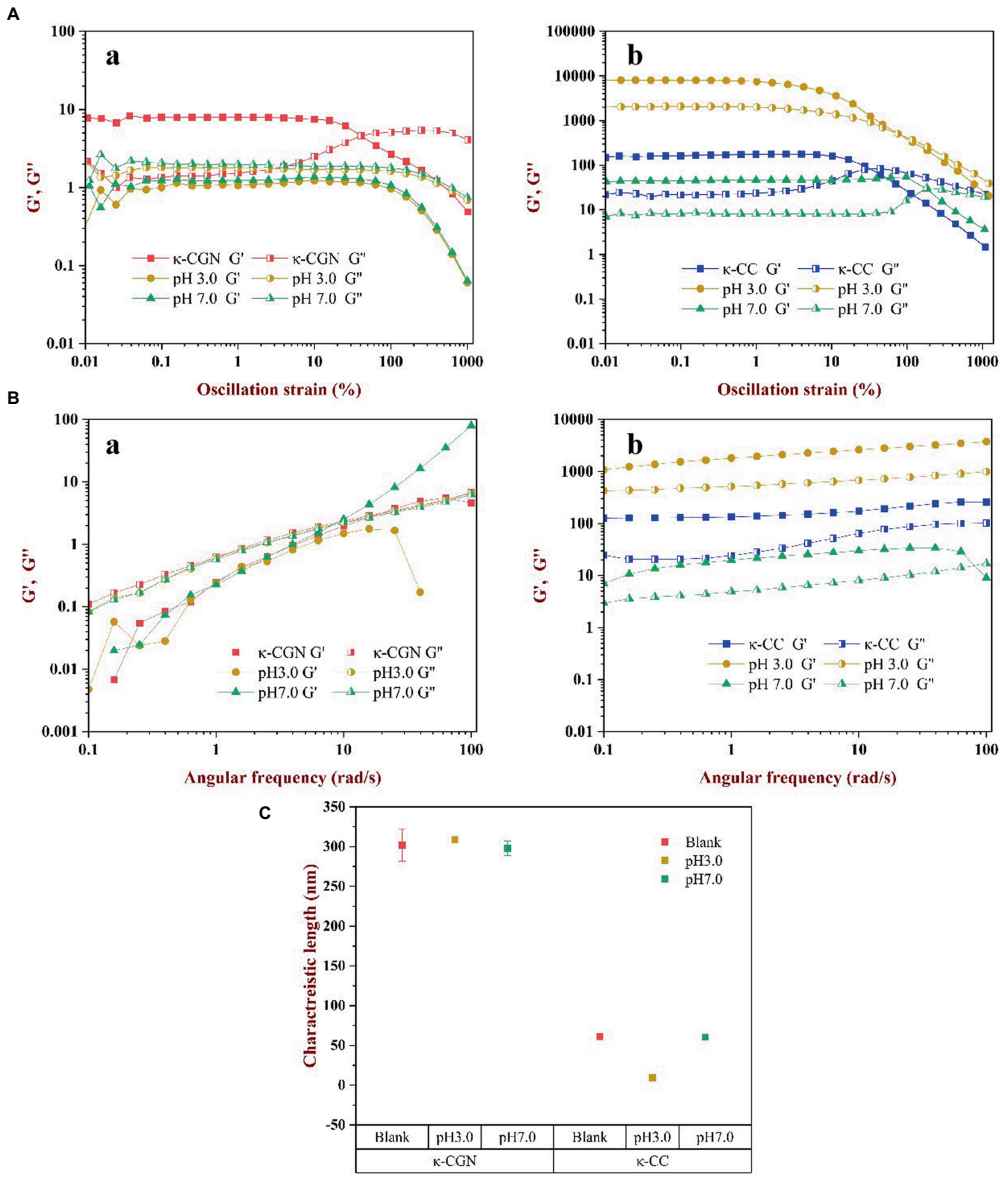
The effects of physiological pH on the rheological characterization of κ-CGN and κ-CC. Dynamic oscillatory measurements were used to show the nonlinear rheology behaviors **(A)**; Frequency sweep test were conducted to analyze the relation of angular frequency and the dynamic moduli **(B)**; The characteristic length of elastically active chain was calculated to reflect the intermolecular elasticity **(C)**.

Substances ensure the integrity of their internal structures in the linear viscoelastic region. Rheological measurements in the linear viscoelastic region can provide meaningful information on the microstructure of complex fluids. The results revealed in the linear viscoelastic region, the values of G′ and G″ were stable (Figure 4A). With strain increasing, G′ and G″ of κ-CGN and κ-CC reached the maximum value before yielding. The modulus decreased sharply, which was due to the destruction of the network structure and the chemical bonds inside the polymer. The spatial structure of the polymer was in a dynamic equilibrium of destruction and recovery in the linear viscoelastic region (31). The strain sweeps of κ-CGN and κ-CC under physiological pH are presented in Figure 4A. The strain range of the linear viscoelastic region of κ-CGN and κ-CC was 0.01–1%. To ensure the relative integrity of the polymer structure, 0.1% strain was selected for frequency sweeping.

#### 3.4.2. Frequency sweep

The frequency sweep of κ-CGN and κ-CC is shown in Figure 4B. In κ-CGN, G″ was greater than G’ during the entire process (Figure 4Ba), indicating that pure κ-CGN was viscous, which was similar with κ-carrageenan/casein mixed gels (32). Figure 4Bb shows the frequency sweep of κ-CC. In this complex, G′ was greater than G″ indicating that the mixed solution had good elastic behavior and formed a weak gel structure. G′ reflected the tightness of the binding between macromolecules, the greater the G′, the tighter the binding between molecules (17). G′ of κ-CC composite system was the largest in pH 3.0 environment, which was mainly attributed to the isoelectric effect of casein. The flocculation and precipitation of casein promote the aggregation of molecular chains, which leads to the enhancement of elastic properties. For polyelectrolyte biopolymers, changes in pH cause changes in the electrical properties and charges on κ-CGN and amino acid residues and affect the interaction between κ-CGN and casein (33). The results revealed that gastric acid environment promoted the intermolecular interaction between κ-CGN and casein, while intestinal environment was in favor of formation loose and orderly helical structure of κ-CC.

#### 3.4.3. The length of elastic active strands

The strength of the interaction between proteins and polysaccharides can be reflected by the elastic activity characteristic length. The smaller the elastic activity characteristic length, the stronger the interaction force and the tighter the structure (34). The elastic activity characteristic length of κ-CGN and κ-CC is shown in Figure 4C. The elastic activity characteristic length of κ-CGN and κ-CC was 288.1 ± 20.3 nm and 31.3 ± 0.8 nm, respectively, indicating that the formation of κ-CGN and κ-CC greatly shortened the distance of intermolecular chains. When the external environmental conditions changed, the characteristic length of the polymers changed, which reflected the dynamic change of its molecular chain. At pH 3.0, the characteristic length of κ-CGN increased from 288.1 ± 20.3 nm to 311.7 ± 2.6 nm, indicating that the distance between molecular chains of κ-CGN increased in acidic conditions, and the random curl conformation increased (18). In contrast, the characteristic length of κ-CC decreased from 31.3 ± 0.8 nm to 9.4 ± 0.8 nm, mainly due to amino protonation occurs in casein under acidic conditions, protein flocculation and precipitation shorten the molecular chain spacing. While the characteristic length of κ-CGN and κ-CC did not change significantly at pH 7.0, demonstrated the orderly conformation.

### 3.5. DSC analysis

**Figure 5:**
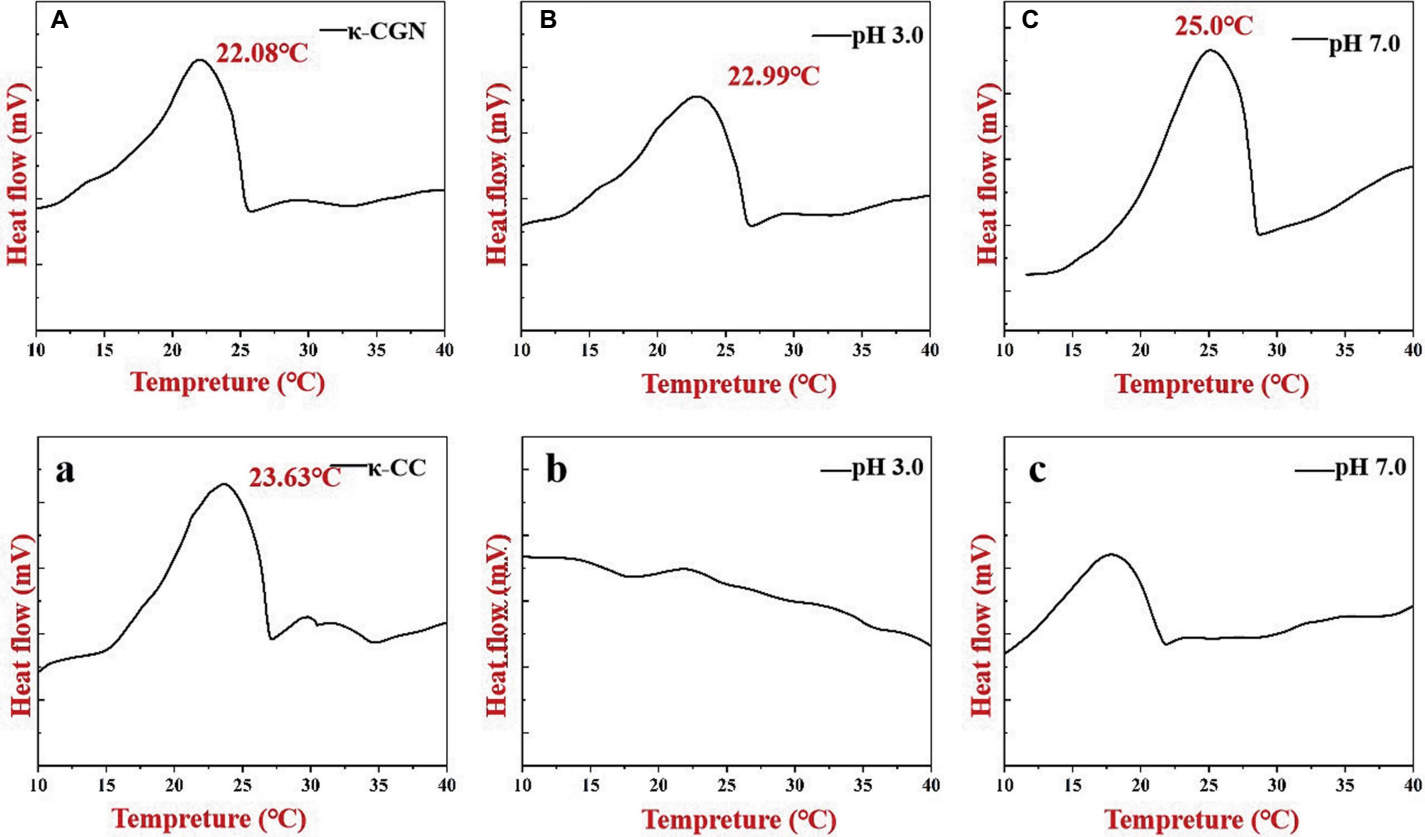
Heat flows of κ-CGN **(A–C)** and κ-CC complexes **(a–c)** at physiological pH in the simulated gastrointestinal phase upon cooling.

Figure 5 shows the thermal hysteresis between the DSC exotherm arising from the disordered-ordered transition upon cooling. We obtained the same exothermic peak during the exothermic process of κ-CGN. The temperature at the top of the exothermic peak can be used to characterize the transition of κ-CGN from a disordered to an ordered conformation (25). Figure 5A shows that the exothermic peak of κ-CGN appeared at 22.08°C, consistent with the results of Zhao et al. (35). The exothermic peaks of κ-CGN at pH 3.0 and pH 7.0 emerged at 22.99 and 25.00°C, respectively, indicating that low pH had little effect on the conformational transition temperature and enthalpy of κ-CGN, while neutral pH can improve the conformational stability of κ-CGN. The exothermic peak of κ-CC during cooling appeared at 23.63°C following the addition of casein (Figure 5A), which was attribute to the order helix conformation transition by the electrostatic interactions and hydrogen bonds between κ-CGN and casein. The higher exothermic peak temperature means higher thermal stability of the gel (36). The DSC results of κ-CC indicated that the electrostatic interaction between casein and κ-CGN increased the gel stability of κ-CC. When the pH of κ-CC was adjusted to 3.0, the exothermic peak of κ-CGN disappeared. At this pH, the protein flocculated and precipitated, which hindered the formation of the κ-CGN gel.

### 3.6. Molecular dynamics simulation

#### 3.6.1. RMSD analysis

**Figure 6:**
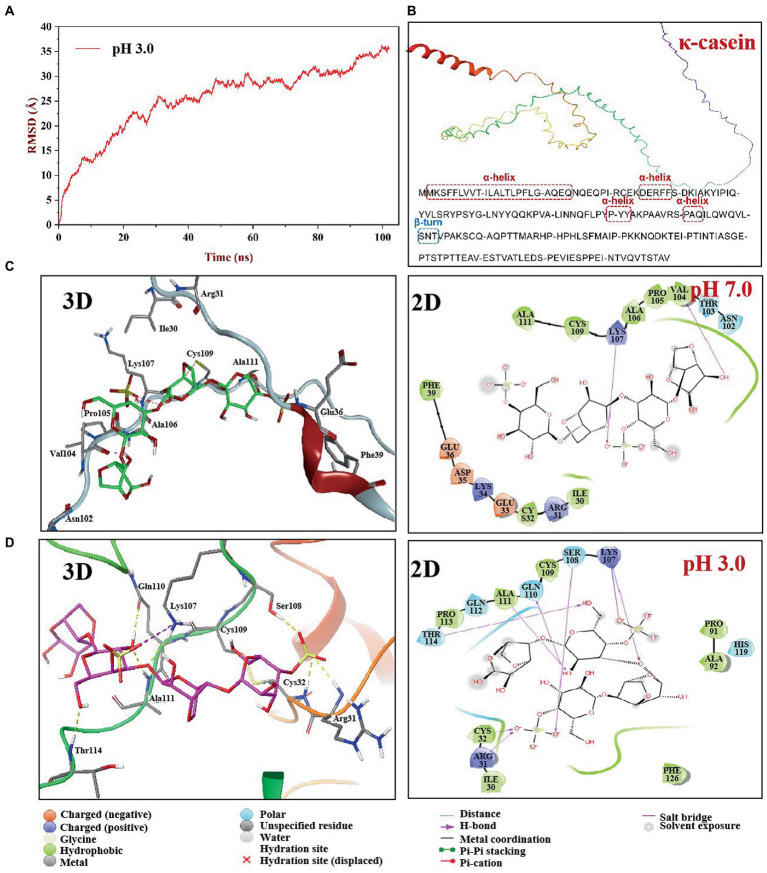
Molecular dynamics simulation of κ-CGN and κ-casein in two pH boxes. The root means square deviation (RMSD) of κ-CGN interaction with κ-casein as a function of simulated times at pH 3.0 condition **(A)**; The 3D structure of bovine κ-casein was automatically figured out by the AlphaFold2 software **(B)**; 3D and 2D interaction plot of κ-CGN and κ-casein at pH 7.0 **(C)** and pH 3.0 **(D)**.

The root mean square deviation (RMSD) is an indicator of whether the simulation system is balanced. The equilibrium state of κ-CC can be assessed by calculating the variation of RMSD. In general, the lower the RMSD, the more stable the simulation system (37). Figure 6A shows that during the 102-ns simulation process, the conformation of κ-CC changed greatly in the first 20 ns. RMSD flattened after 30 ns of simulation, indicating that the system had gradually reached equilibrium state. We selected the trajectory of the equilibrium phase for subsequent interaction and conformational analyses.

#### 3.6.2. Interaction and conformational analysis

To understand the effect of physiological pH environment during gastrointestinal digestion on the interaction between κ-CGN and casein, we analyzed the structural changes of κ-CC at pH 3.0 and pH 7.0 solvent boxes. The κ-casein was located on the periphery of casein micelle, providing a steric and electrostatic stabilizing outer layer, which bind with κ-CGN through the positively charged amino acids of κ-casein and the negatively charged sulfated groups of κ-CGN (38). Therefore, the molecular model of bovine κ-casein was built to analyze the biding sites with κ-CGN using the AlphaFold2 software (Figure 6B). In neutral pH 7.0 solvent box, κ-casein formed an α-helix in amino acid fragments 2–24, 35–39, and 90–94 (17.37%), a short β-turn in fragment 101–102 (0.01%), and the remainder fragments formed a flexible coil area (81.05%). In pH 3.0 solvent box, the rigid region α-helix structure and flexibility region coil structure were partially transformed into β-Sheet and β-turn structures, and the flexible coil structure content decreased from 81.05 to 66.84% (Table 1), indicating that the fewer flexible regions, stronger rigidity structures, and stronger intermolecular hydrogen bonding between κ-casein and κ-CGN. Additionally, binding free energy assessment was carried out to analyze the binding site location and binding ability of κ-CGN to κ-casein. As the results showed, absolute value of binding free energy increased from 7.23 kcal mol^−1^ (pH 7.0) to 8.28 kcal mol^−1^ (pH 3.0), indicating that the higher binding sites were occurred in pH 3.0 solvent box.

**Table 1:** The secondary structure content of κ-casein at physiological pH (%).

	α-Helix	β-Sheet	Coil	Turn
MD pH 7.0	17.37	0.00	81.05	0.01
MD pH 3.0	11.05	6.32	66.84	15.79

In the pH 7.0 solvent box (Figure 6C), the κ-CGN chains were quasi-rigid, appearing a rod-like shape with no sharp bending (39). κ-CGN was surrounded by the amino acid residues of κ-casein: Ala111, Cys109, Lys107, Ala106, Pro105, Val104, Asn102, Phe39, Glu36, Asp35, Lys34, Glu33, Cys32, Arg31, and Ile30. Additionally, the pattern of solute-solute hydrogen bonds was observed on -OH groups of κ-CGN with Val104 and on -SO_3_^−^ group with Lys107 of κ-casein. Meanwhile, the -SO_3_^−^ group at one end of κ-CGN formed electrostatic interactions with Lys107 of κ-casein. The van der Waals interactions in the binding sites of Ile30, Phe39, Ala106, and Glu36 were observed. The -SO_3_^−^ group at the other end was exposed to the solvent without binding with κ-casein, surrounded by polar amino acids Glu36, Lys34, and Asp35. In pH 3.0 solvent box, the rigid conformation of κ-casein was enhanced by the transformation of flexibility region coil structure into a sheet structure, and the folds and helical shapes of κ-CGN in a dimensional scale were observed, both which might result in the increase of binding sites between κ-CGN and κ-casein (Figure 6D). A -SO_3_^−^ group at one end of κ-CGN formed salt bridges with Lys107 of κ-casein, and hydrogen bonds on the other -SO_3_^−^ group (with Arg31, Ser108, and Cys32) and OH- groups (with Lys107, Gln110, Thr114, and Ala111) were identified. Additionally, the electrostatic interactions with Arg31 and Lys 107 were observed.

The results of molecular dynamics simulation demonstrated that gastric acid environment promoted secondary structure of κ-casein partially transformed from flexibility coil structures into rigidity structures, leading the binding sites between κ-CGN and κ-casein increased. Seven hydrogen bonds (Arg31, Ser108, and Cys32 on -SO_3_^−^ group, Lys107, Gln110, Thr114, and Ala111 on OH- group) were observed in pH 3.0 solvent box, while two hydrogen bonds (Val104 on -SO_3_^−^ group, and Lys107 on OH- group) were observed in pH 7.0 solvent box. The increased binding sites expressed on the -SO_3_^−^ groups of κ-CGN also lead to the exposure of free -SO_3_^−^ groups declined in intestinal environment (pH 7.0).

## 4. Conclusion

The structure and solution behavior of polysaccharides are closely related to their biological activity. Studies had reported that vehicle (aqueous solution or protein solvent) might affect the colitis of κ-CGN, due to it could influence their conformation changes and the exposure of free -SO_3_^−^ groups. This study focused on the effects of pH in the gastrointestinal tract on κ-CGN and κ-CC, conformational transitions and exposure of free -SO_3_^−^ groups. In gastric acid environment (pH 3.0), the charge density on the surface of κ-CGN molecular chains was decreased, resulting in a decrease in conformational stability, accompanied by the increased exposure of sulfuric acid groups, which lead to colitis risk increase of κ-CGN. In contrast, κ-CC was much more stable in gastric acid environments due to casein flocculation caused secondary structure folding, thereby enhanced the intermolecular electrostatic interactions between κ-CGN and casein, and significantly reduced the exposure of free sulfate groups of κ-CGN. These results were confirmed by molecular dynamics simulations. It can be found that under the condition of pH 3.0 solvent box, the molecular chains of κ-CGN folded with the ordered conformation of κ-casein structure, at the same time, the molecular chains’ spacing decreased, and the interaction sites between κ-CGN and κ-casein increased. However, in the intestinal box (pH 7.0), κ-CGN and κ-CC did not change much, indicating that their conformation is relatively stable in neutral environment. Our research results provide an important theoretical basis for elucidating the molecular mechanism and structure–activity relationship of κ-CGN under casein matrix to protect the mucosal barrier and inhibit colitis, and are of great significance for guiding and expanding the safe application of κ-CGN.

## Data availability statement

The original contributions presented in the study are included in the article/Supplementary material, further inquiries can be directed to the corresponding author.

## Author contributions

JG designed the work, and revised it critically for important intellectual content, and agreed to be accountable for all aspects of the work in ensuring that questions related to the accuracy or integrity of any part of the work are appropriately investigated and resolved. SZ, PC, and LL designed the work, acquisition, analysis, and interpretation of data for the work. ZL provided approval for publication of the content. JZ revised the work critically for important intellectual content. All authors contributed to the article and approved the submitted version.

## Funding

This work was supported by the National Natural Science Foundation of China (No. 32001625), Open Project of Key Laboratory of Cultivation and High-Value Utilization of Marine Organisms in Fujian Province (No. 2022fjscq03), Fujian Province Key Laboratory for the Development of Bioactive Material from Marine Algae (No. 2022KF09), Fujian Provincial Education Department of young and middle-aged teachers Education research project (No. JAT190521), and Natural Science Foundation of Fujian Province (No. 2020J01787).

## Conflict of interest

The authors declare that the research was conducted in the absence of any commercial or financial relationships that could be construed as a potential conflict of interest.

## Publisher’s note

All claims expressed in this article are solely those of the authors and do not necessarily represent those of their affiliated organizations, or those of the publisher, the editors and the reviewers. Any product that may be evaluated in this article, or claim that may be made by its manufacturer, is not guaranteed or endorsed by the publisher.

## Footnotes

^1^ https://pubchem.ncbi.nlm.nih.gov/
